# Comparison of Serum Neutrophil Gelatinase-associated Lipocalin (NGAL) with Serum Creatinine in Prediction of Kidney Recovery after Renal Transplantation

**Published:** 2012-11-01

**Authors:** M. Mahdavi-Mazdeh, M. Amerian, A. Abdollahi, Z. N. Hatmi, M. R. Khatami

**Affiliations:** 1 *Iranian Tissue Bank Research and Preparation Center, *; 2 *Research Center of Nephrology, *; 3 *Department of Social Medicine, Tehran University of Medical Sciences, Tehran, Iran*

**Keywords:** Transplantation, Dialysis, Serum creatinine, Lipocalins, Serum neutrophil gelatinase-associated lipocalin

## Abstract

**Background:** Because of some insult to kidney during transplantation, assessment of kidney function after the procedure is essential. It would be ideal to find a marker better than creatinine to early predict the acute kidney injury.

**Objective:** To compare with creatinine the predictive value of serum neutrophil gelatinase-associated lipocalin (NGAL) in detecting kidney recovery after renal transplantation.

**Methods:** We studied 33 patients who received kidney transplantation (deceased [n=20] and live [n=13]) during a 6-month period in 2010. Serum NGAL and creatinine, hemoglobin, and blood glucose were measured at 0, 12, 24, 48, and 72 hours after transplantation. The need for dialysis and kidney function in one week were studied.

**Results:** There were 16 men and 17 women with the mean±SD age of 36.3±12.2 (range: 14–58) years. Of the studied patients, 6 had delayed graft function (DGF; hemodialysis within the first week of transplant); 9 had slow graft function (SGF; serum creatinine reduction from transplantation to day 7 <70%), and 23 had immediate graft function (IGF; reduction in serum creatinine ≥70%). At any time, serum NGAL, and creatinine levels were significantly higher among patients with DGF (p=0.024) and SGF (p=0.026) compared with those with IGF. However, in those who got IGF *vs* non-IGF, serum creatinine levels were not significantly different (p=0.59) but serum NGAL levels differed significantly(p=0.020). Receiver-operating characteristic (ROC) curve and area under curves (AUCs) of serum NGAL and serum creatinine levels on the first post-transplantation day had similar significance in predicting the patient’s need to dialysis in the first week. However, using AUC of serum creatinine was not helpful in predicting non-IGF, compared to serum NGAL. The AUCs of the serum NGAL were 0.70 (95% CI: 0.52–0.89) and 0.76 (95% CI: 0.59–0.93) after 12 and 24 hours, respectively (p<0.05). The highest AUC (0.82) was attributed to serum NGAL of 24 hour (p=0.002).

**Conclusion:** Serum NGAL level especially 24 hours post-transplantation, seems to be an early accurate predictor of both the need to dialysis and slow graft function within the first week of kidney transplantation.

## INTRODUCTION

Acute kidney injury (AKI) is a common problem in many clinical settings including renal transplantation. Because of many limitations, indicators such as serum creatinine or urinary output are not reliable for early diagnosis of deteriorating renal function. A biomarker to facilitate prediction of kidney function would be valuable. Based on published articles, it seems that urinary and plasma neutrophil gelatinase-associated lipocalin (NGAL), are reliable biomarkers for the subsequent development of clinical AKI. However, there is not a consensus on this biomarker for prediction of graft dysfunction after transplantation [1-4].

Belonging to the lipocalin superfamily, NGAL, a 25-kDa protein was initially found in activated neutrophils. However, other cells, such as renal tubular cells, may release NGAL in response to inflammation or injury [5,6].

In a meta-analysis of 19 studies with 2538 patients, it has been documented that NGAL level increases in urine and/or plasma before clinical diagnosis of AKI with prognostic significance; it has also been shown to be a useful indicator to predict renal replacement therapy initiation and in-hospital mortality [7].

In kidney transplantation, Mishra, *et al*., examined 25 specimens of kidneys biopsies obtained at approximately one hour of reperfusion after transplantation for NGAL expression by immunohistochemistry and found a strong correlation between NGAL expression not only with cold ischemia time (r=0.87, p<0.001) but also with peak post-operative creatinine level days later (r=0.86, p<0.001) [3]. NGAL level in post-transplantation period has positive correlation with serum creatinine; the first day urinary NGAL predicts dialysis need within the first week of transplantation better than serum creatinine [2,8,9] Hollman, *et al*., in their study on 176 kidney recipients, also showed slower decrease in urinary NGAL compared to those without delayed graft function (DGF); urinary NGAL of the first post-transplantation day predicted DGF even when the clinicians did not expect which reflects the prognostic value of NGAL in these patients [10]. The value of NGAL was even highlighted in prediction of early functional recovery of transplanted kidneys from cardiac death donors in comparison with living donors [11].

The objective of this study was to re-evaluate the predictive value of serum NGAL in predicting DGF in kidney transplantation and figure out the best time for measurement of NGAL.

## PATIENTS AND METHODS

This prospective observational study was performed on 33 renal allograft recipients who received their kidneys in Imam Hospital, Tehran, Iran, from March 2010 to September 2010. The immunosuppressive regimen consisted of cyclosporine, mycophenolate mofetil, and prednisolone. The protocol of this study was approved by the Medical University Ethics Committee. Serum NGAL (Antibodyshop, Gentofte, Denmark) and creatinine, hemoglobin, blood glucose (commercially available kits) and urine output were measured at 0, 12, 24, 36, 48 and 72 hours after transplantation. Serum creatinine was measured by Jaffé method. Routinely, all patients underwent renal scintigraphy on the second day after operation. Measurements were performed by hospital laboratory personnel who were not aware of the NGAL source.

We used the definition of Hall, *et al*, for DGF *(i.e.,* need to dialysis in the first week of transplantation), slow graft function (SGF, *i.e.*, reduction in serum creatinine on day seven to the time of transplantation <70%), and immediate graft function (IGF, *i.e.*, >70% reduction of serum creatinine on seventh day in comparison with transplantation time) [12].

Statistical analysis

Data were expressed as mean±SD (range). One-way analysis of variance (ANOVA) was used to assess the level of plasma NGAL at different hours in three levels of graft function. Kruskall-Wallis test was used to determine if the difference in cause of renal failure, sex of donor or recipient had any impact on renal function after transplantation. Repeated-measures ANOVA was used for plasma NGAL and creatinine levels at different time points. Correlations between NGAL and other variables were evaluated by Pearson’s or Spearman’s correlation coefficients, as appropriate. Receiver-operating characteristic (ROC) curve was used to determine the cutoff point for plasma NGAL level to predict DGF. All tests were two-sided; p<0.05 was considered statistically significant.

## RESULTS

The study was conducted on 33 kidney recipients. There were 16 men and 17 women. The mean±SD age of participants was 36.3±12.2 (range: 14–58) years. In 12 (36%) patients the cause of end-stage renal disease (ESRD) was unknown. Hypertension and diabetes were the cause in 11 (33%) and 4 (12%) patients, respectively. Four (12%) patients had preemptive transplantation and the rest had history of dialysis. The source of kidney was from brain-dead donors (BDD) in 20 (61%) patients.

Twenty-seven (82%) patients were prescribed polyclonal antibodies during induction therapy. The demographic data are shown in different groups in Table 1.

**Table 1:** Recipients and donors characteristics

Parameter	All (n=33)	DGF (n=6)	SGF (n=8)	IGF (n=19)	p value
Source of kidney					0.60
BDD LD	20 13	3 3	6 2	11 8	
Mean±SD (range) recipient age (yr) BDD LD	36.3±12.2 (14–58) 36±12.7 36.8±11.9	36.0±12.7 29.0±9.5 43.7±8.0	37.9±14.3 39.2±15.1 35.0±21.2	35.5±11.7 36.2±12.4 34.6±11.5	0.89
Recipient sex (M/F) BDD LD	10/10 6/7	2/3 2/1	4/5 1/5	10/9 7/4	0.15
ESRD due to DM BDD LD	2/20 2/13	1/6 0 1	1/7 1 0	2/17 1 1	0.92
Mean±SD daily urine volume before transplantation (mL) BDD LD	197.5±334.2 50±86.6	220±438 333.3±577.4 133.3±152.8	267±346.4 350.0±393.7 0	57.9±139.7 77.3±180.8 31.3±45.8	0.15
Dialysis before transplantation (yes/no) BDD LD	16/4 13/0	5/0 3/0 3/0	6/3 3/3 2/0	18/1 10/1 8/0	0.84
Mean±SD hemodialysis duration (m) BDD LD	30.2±31.7 30.2±36.7 30.3±23.2	50.7±58.8	15.8±17.8	29.9±21.6	0.12
Donor sex (M/F) BDD LD	16/4 11/2	4/1 2/1 3/0	7/2 4/2 2/0	16/3 10/1 6/2	0.40
Mean±SD donor age (yr) BDD LD	27.9±9.0 (16–55) 29.25±11.1 25.77±3.47	30.7±8.4	34.8±12.1	24.1±5.3	0.009
Polyclonal antibodies (yes/no) BDD (yes) LD (yes)	27 18 9	6/0 3 3	5/3 4 1	16/3 11 5	0.276
Mean±SD (range) cold ischemia time (min) BDD LD	58±54 (10–240) 84.25±55 17.7±8.80	46.7±42.6	73.1±52.8	55.3±58.8	0.64
Mean±SD warm ischmia time (min) BDD LD		105.0±231.5	128.8±37.2	97.9±22	0.05

Serum NGAL level did not show significant difference between sex of recipients or history of hypertension/diabetes. It also did not have any correlation in separate analysis of BDD and LD recipients, with cold and warm ischemia time. At each time, serum NGAL was correlated positively to serum creatinine level. Serum NGAL level on the first day and its rate of decrease after transplantation were significantly different among the studied groups (p=0.014 and 0.003, respectively); however, it was not significantly different among the groups before transplantation. The serum level of NGAL after 12 hours of transplantation had a significant (p<0.001) correlation with renal scan findings; the level after the operation correlated negatively with urine volume during the same period (r = 0.34, p=0.05). Serum creatinine level after 48 hours of transplantation had significant predictive value for kidney function (24 hrs, p=0.15; 48 hrs, p=0.04).

**Table 2:** Comparison of kidney function recovery during post-transplantation period. Numbers are mean±SD.

Marker	DGF (n=5)	SGF (n=9)	IGF (n=19)	p value
NGAL0 Creat0	6500±1542.7 7.3±1.0	4255.6±2631.1 5.5±1.9	3826.3±2650.7 7.2±1.5	0.125 0.29
NGAL12 Creat12	4580.0±2507.4 6.1±1.3	3644.4±2112.0 4.2±1.6	2684.2±1962.0 5.0±1.5	0.170 0.74
NGAL24 Creat24	4120.0±2232.0 5.6±1.2	3011.1±1832.6 3.6±1.4	1721.1±1387.1 3.9±1.1	0.014 0.15
NGAL48 Creat48	3360.0±2250.1 4.1±1.9	2300.0±1572.4 2.8±1.3	1205.8±889.2 2.5±0.8	0.007 0.04
NGAL72 Creat72	2916.0±2147.6 3.5±1.9	2067.8±1523.6 2.4±1.1	736.8±618.3 1.7±0.7	0.001 0.04
Ratio NGAL 12/0 Creat 12/0	0.66±0.23 0.83±0.1	0.86±0.15 0.78±0.1	0.71±0.15 0.70±0.1	0.06 0.05
Ratio NGAL 24/0 Creat 24/0	0.59±0.23 0.77±0.15	0.70±0.24 0.65±0.1	0.45±0.13 0.55±0.13	0.003 0.005

Urine output after operation had a significant negative correlation with creatinine level at 12, 24, 48 and 72 hours of transplantation; however, its negative correlation lasts till 24 hours after transplantation with serum NGAL level. At any time, serum NGAL, and creatinine levels were significantly higher among patients with DGF and SGF compared with those with IGF (p=0.024 and 0.026, respectively). However, when they were compared between those who got IGF *vs*. non-IGF, serum creatinine levels were not significantly different (p=0.59) and serum NGAL levels differed significantly (p=0.02).

**Figure 1:**
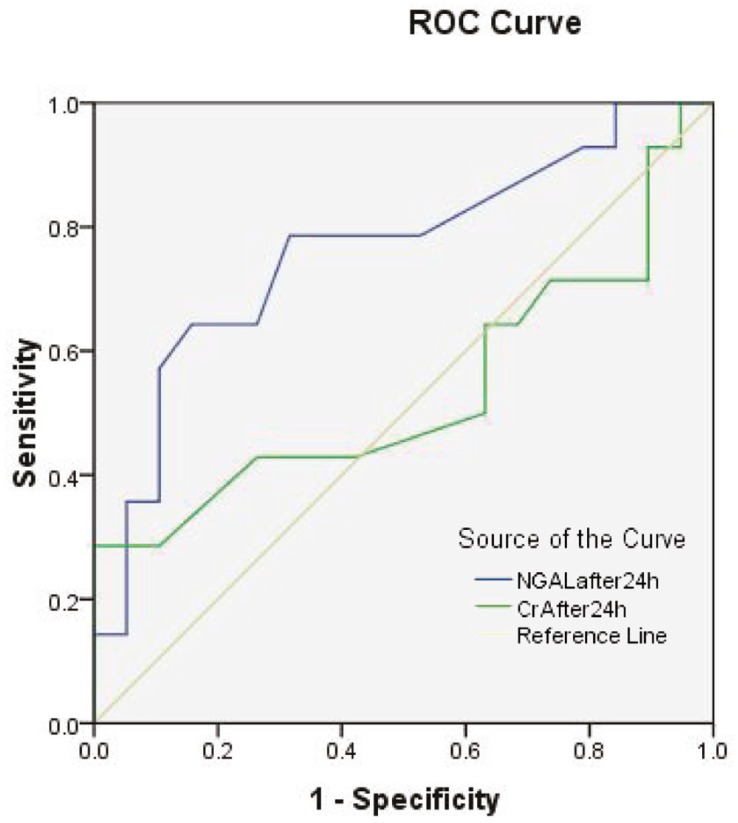
ROC curves of NGAL and creatinine at 24 hours of transplantation

In order to assess the predictive value of NGAL *vs*. creatinine for DGF or non-IGF (combined DGF or SGF) we compared the areas under the ROC curves (AUCs) (Table 3). Serum NGAL or serum creatinine on the first post-transplantation day had similar AUC and predictive value to predict the patient’s need to dialysis in the first week. However, using the AUCs of serum creatinine was not helpful in comparison with serum NGAL for predicting non-IGF. The AUCs of the serum NGAL were 0.70 (95% CI: 0.52–0.89) after 12 hours and 0.76 (95% CI: 0.59–0.93) after 24 hours (p<0.05). The highest AUC was attributed to serum NGAL of 24 hours (0.82, p=0.002) (Fig. 1).

**Table 3:** ROC of NGAL and creatinine levels to predict DGF or non-IGF

Marker *vs* time	DGF		Non-IGF	
	AUC (95% CI)	p value	AUC (95% CI)	p value
After operation NGAL (ng/mL) -BDD -LD Creat	0.80 (0.66–0.94) 0.82 (0.65–1.00) 0.88 (0.69–1.08) 0.53 (0.30–0.76)	0.02^*^ 0.08 0.05 0.85	0.68 (0.50–0.87) 0.34 (0.13–0.54)	0.08 0.11
12 hours NGAL -BDD -LD Creat	0.74 (0.55–0.92) 0.82 (0.64–1.01) 0.73 (0.46–1.00) 0.71 (0.50–0.92)	0.08 0.08 0.24 0.11	0.70 (0.52–0.89) 0.49 (0.27–0.70)	0.05* 0.89
24 hours NGAL -BDD -LD Creat	0.80 (0.59–1.0) 0.87 (0.70–1.04) 0.78 (0.41–1.15) 0.80 (0.61–0.99)	0.02* 0.04* 0.15 0.03*	0.76 (0.59–0.93) 0.54 (0.32–0.75)	0.01* 0.73
48 hours NGAL -BDD -LD Creat	0.85 (0.68–1.0) 0.92 (0.79–1.05) 0.82 (0.54–1.09) 0.80 (0.55–1.1)	0.009* 0.02* 0.11 0.02*	0.78 (0.61–0.94) 0.63 (0.41–0.84)	0.007* 0.22
72 hours NGAL -BDD -LD Creat	0.83 (0.61–1.0) 0.92 (0.79–1.05) 0.77 (0.37–1.16) 0.82 (0.55–1.1)	0.01* 0.02* 0.18 0.02*	0.82 (0.65–0.98) 0.70 (0.50–0.90)	0.002* 0.05

LRD: Living related donor; LURD: Living unrelated donor

AUCs for predicting DGF and non-IGF using serum creatinine on the first post-operative day were 0.80 (95% CI: 0.61–0.99) and 0.54 (95% CI: 0.32–0.75), respectively.

## DISCUSSION

Diagnosis of acute graft dysfunction is usually made with increased serum creatinine concentration, which is a late signal of kidney injury. Nevertheless, the sooner treatment is initiated the better outcome is expected. The treatment should be as close as possible to the insult just before the diagnosis is made by serum creatinine [13].

Our study, like other studies, confirmed that plasma NGAL level was more useful than absolute or percentage of serum creatinine decrease in predicting DGF [7,9,11,12]. However, plasma NGAL level before with first 24 hours of transplantation was not able to differentiate patients with DGF from those with non-DGF. In this study, we found that the best time for measuring plasma NGAL level for such purpose is 24 hours after the transplantation. Similar to Lebkowska, *et al*., we found a significant decrease in serum NGAL as early as one day after transplantation—before the serum creatinine level decreases [2]. Bataille, *et al*., in 41 transplant patients found an early and significant post-operative decrease in plasma NGAL after 12 hours of transplantation in the IGF group, which remained elevated in the DGF group throughout the follow-up period [14]. Similar to our study, they showed that the decrease in creatinine level was delayed compared with plasma NGAL decrease.

Mishra, *et al*., found the highest NGAL staining intensity in biopsies of one hour of reperfusion after transplantation in those with DFG [3]. Although plasma NGAL level after transplantation was highest in DGF group, surprisingly, no significant correlation was observed between NGAL and DGF, which may be due to limited sample size or its excretion into the urine rather than being released into the circulation. It was shown that the urine output after operation negatively correlated with plasma NGAL after operation which supports the explanation.

The main limitation of our study was the small sample size (20 from BDD) for which we could not do subgroup analysis. However, based on prior studies, the results support the finding that plasma NGAL level, especially after 24 hours of transplantation, is an early accurate predictor of both the need to dialysis and slow graft function within the first week of kidney transplantation.
